# Navigating structural and personal dilemmas: a phenomenological study of midwives providing legal abortion care in Nampula, Mozambique

**DOI:** 10.1080/26410397.2026.2637328

**Published:** 2026-03-04

**Authors:** Gilda Gondola Sitefane, Khátia Munguambe, Pia Axemo, Johanna Belachew, Birgitta Essén, Esmeralda Mariano

**Affiliations:** a PhD Candidate, Department of Women and Children's Health, International Maternal Health, and Migration Unit (IMHm), Uppsala University, Uppsala, Sweden.; b Auxiliary Professor, Department of Community Health, Sexual and Reproductive Health Unit, Faculty of Medicine, Eduardo Mondlane University, Maputo, Mozambique; c Auxiliary Professor, Department of Women and Children's Health, International Maternal Health, and Migration Unit (IMHm), Uppsala University, Uppsala, Sweden; d Senior Lecturer, Department of Women and Children's Health, International Maternal Health, and Migration Unit (IMHm), Uppsala University, Uppsala, Sweden; e Professor, Department of Women and Children's Health, International Maternal Health, and Migration Unit (IMHm), Uppsala University, Uppsala, Sweden; f Auxiliary Professor, Department of Archaeology and Anthropology, Faculty of Arts and Social Sciences, Eduardo Mondlane University, Maputo, Mozambique

**Keywords:** abortion, young pregnant women, midwives, qualitative study, phenomenology, intersectionality, Mozambique

## Abstract

Legal reform allowing abortion is often considered a proxy for the provision of services. While the law establishes a legal framework, the provision of services is closely tied to the engagement of midwives in service delivery. In 2014, Mozambique decriminalised abortion under specific conditions. Studies of this legal reform focus on the experience of pregnant women, leaving the experience of midwives largely unexplored. This study focuses on midwives providing abortion services to young women (ages 15–24). A qualitative phenomenological study was conducted in Nampula province, northern Mozambique. In-depth interviews were conducted with 10 purposively selected midwives across six primary healthcare facilities in three districts. Inductive thematic analysis was employed, and an intersectionality lens guided the discussion. We employed continuous reflection and adhered to COREQ guidelines for the conduct, analysis and reporting of this study. Despite general support among midwives for abortion service availability and provision, some expressed distress and objections to providing the service themselves. A complex interplay between structural and personal factors was significant in shaping their experiences. Traumatic encounters with abortion-related complications emerged as the central factor behind their distress and objections. The intersections of personal factors with structural dilemmas, including a few health facilities authorised to provide services, a lack of abortion medicines, and legal restrictions for adolescents under age 16, shaped their professional experience, often leading to work-related distress, and challenged their willingness to provide services. Policy efforts should address these barriers, prioritising expansion of service provision, alignment with human rights standards, and midwife training and support.

## Plain language summary

Changes in the laws regarding abortion are often seen as a sign that safe abortion services are available. In 2014, Mozambique changed its law to allow abortion in certain situations. While most research since then has focused on how the law affects women seeking abortions, little attention has been given to the experiences of midwives who play a key role in service provision. In this study, we aimed to understand midwives’ experiences in providing legal abortion services, especially to young women aged 15–24, who are most at risk of unsafe abortion and maternal mortality. Researchers interviewed 10 midwives working in health facilities in Nampula Province, Mozambique. Although midwives generally supported abortion service availability and provision, some had been through traumatic experiences managing unsafe abortion complications, including deaths. This created emotional distress and affected their willingness to continue to provide abortion services. Structural difficulties facing midwives included the limited number of health centres authorised to provide abortion care, frequent shortages of essential medicines, and strict legal requirements for helping girls under 16. These challenges affected both the midwives’ well-being and the care they could provide. This paper recommends that policies address these barriers by: (1) increasing the number of approved health facilities, (2) aligning abortion services with human rights standards (especially for adolescents), and (3) offering more training and emotional support for midwives in providing abortion care.

## Résumé

La réforme juridique autorisant l'avortement est souvent considérée comme un indicateur de la prestation de services. Si la loi établit un cadre légal, la prestation de services est étroitement liée à la participation des sages-femmes. En 2014, le Mozambique a dépénalisé l'avortement sous certaines conditions précises. Les études portant sur cette réforme se concentrent sur l'expérience des femmes enceintes, laissant largement inexplorée celle des sages-femmes. Cette étude s'intéresse aux sages-femmes pratiquant l'avortement chez des jeunes femmes (âgées de 15 à 24 ans). Une étude phénoménologique qualitative a été réalisée dans la province de Nampula, au nord du Mozambique. Des entretiens approfondis ont été menés auprès de 10 sages-femmes choisies à dessein dans six centres de soins de santé primaires répartis dans trois districts. Une analyse thématique inductive a été employée alors qu'un éclairage intersectionnel a guidé la discussion. Nous avons utilisé une approche réflexive continue et respecté les lignes directrices COREQ pour la conduite, l'analyse et la rédaction de cette étude. Même si les sages-femmes soutiennent en général l'accès à l'avortement, certaines ont exprimé leur désarroi et des réticences à pratiquer elles-mêmes ces avortements. Une interaction complexe entre les facteurs structurels et personnels a joué un rôle important pour façonner leurs expériences. Les expériences traumatisantes liées aux complications de l'avortement sont apparues comme la principale source de leur désarroi et de leurs objections. La conjugaison de facteurs personnels et de dilemmes structurels, notamment le faible nombre d'établissements de santé autorisés à pratiquer l'avortement, le manque de médicaments abortifs et les restrictions légales pour les adolescentes âgées de moins de 16 ans, a modelé leur expérience professionnelle, engendrant souvent une souffrance liée au travail et remettant en question leur volonté d'exercer. Les mesures politiques devraient s'attaquer à ces obstacles, en donnant la priorité à l'élargissement de l'offre de services, l'alignement sur les normes relatives aux droits humains ainsi que la formation et le soutien des sages-femmes.

## Resumen

La reforma legislativa para permitir el aborto a menudo es considerada como indicador de la prestación de servicios. Aunque la ley establece el marco legislativo, la prestación de servicios está estrechamente ligada a la participación de las parteras en proporcionar esos servicios. En 2014, Mozambique despenalizó el aborto bajo ciertas condiciones espe­cíficas. Los estudios de esta reforma legislativa se centraron en la experiencia de mujeres embarazadas, sin casi explorar la experi­encia de las parteras. Este estudio se centra en parteras que proporcionan servicios de aborto a mujeres jóvenes (de 15 a 24 años). Se realizó un estudio fenomenológico cualitativo en la provincia de Nampula, en el norte de Mozambique. Se realizaron entrevistas a profundidad con 10 parteras seleccionadas deliberadamente en seis centros de atención primaria en tres distritos. Se empleó el análisis temático inductivo, y un enfoque interseccional guió el debate. Empleamos reflexión continua y nos adherimos a las directrices COREQ para la ejecución, el análisis y la rendición de informes de este estudio. A pesar de que las parteras en general apoyaban la disponibilidad y prestación de servicios de aborto, algunas expresaron angustia y objeciones a proporcionar los servicios ellas mismas. Una interacción compleja entre los factores estruc­turales y personales desempeñó un papel significativo en moldear sus experiencias. Encuentros traumáticos con complica­ciones relacionadas con el aborto surgieron como el factor central detrás de la angustia y objeciones de las parteras. Las intersecciones de factores personales con dilemas estructurales, tales como nuevos centros de salud autorizados para proporcionar servicios, falta de medicamentos abortivos y restricciones legislativas para adolescentes menores de 16, moldearon su experiencia profesional, y a menudo les causaban angustia relacionada con su trabajoy les resultaba más difícil estar dispuestas a proporcionar los servicios. Los esfuerzos de política deberían abordar estas barreras y priorizar la ampliación de la prestación de servicios, la alineación con las normas de derechos humanos, y la capacitación y el apoyo brindados a las parteras.

## Introduction

Unintended pregnancy remains a significant public health concern globally, with abortion being a common outcome,^1,2^ including in Mozambique. For many women, unsafe abortion, performed using unconventional methods not tested or approved by the World Health Organization,^3,4^ remains their only option, contributing 13–18% of global maternal deaths annually.^5^ Nearly all abortion-related deaths (97%) occur in low- and middle-income countries, with Africa disproportionately bearing 63% of these fatalities, ranking the major cause of maternal mortality among adolescent girls and young women aged 15–24.^6^

Mozambique is a low-income country located in Sub-Saharan Africa^7^ with a maternal mortality ratio of 233 per 100,000 live births.^8^ In the early 2000s, unsafe abortion accounted for 11% of maternal deaths in the country.^8^ Of the total maternal deaths, 65% occurred among young women aged 15–30.^9,10^ In 2014, recognising international evidence on the role of legal reforms in reducing abortion-related mortality,^11,12^ driven by national data highlighting the burden of unsafe abortion-related deaths,^9,10,13,14^ Mozambique revised its inherited colonial-era Penal Code dating from 1886. The 1886 penal code criminalised abortion, imposing a 2–8 year prison sentence on both the woman and the healthcare provider.^15^

The 2014 penal code stipulates that voluntary termination of pregnancy* (henceforward, simply “abortion”) is not a crime, provided the following conditions are fulfilled: (1) performed with the pregnant woman’s consent, (2) in a health facility, by a qualified health professional, (3) within the first 12 weeks of pregnancy for any reason, (4) or up to the 16th week in case of rape or incest, (5) the 24th week in case of fetal impairment, and (6) for girls under 16, an adult witness consent is required. In all cases, at public health facilities, it must be provided free of charge, whether using pharmacological or surgical methods. In 2017, following the law’s enactment, the Ministry of Health (MoH) approved its respective clinical standards.^16^ Accordingly, all health facilities can provide post-abortion care. However, a health facility must be formally accredited by the MoH to offer abortion. Otherwise, it must ensure referral to an accredited facility. Accreditation requirements include adequate infrastructure ensuring privacy, trained personnel on abortion regulation and clinical standards and procedures, and necessary equipment, medicines, and supplies. If the requirements are fulfilled, any health facility, including the primary healthcare centres, can provide abortion up to 12 weeks. Despite services being expected to be provided at all levels of care and by different cadres, abortion up to 12 weeks’ gestational age is primarily provided at primary healthcare level, by midwives, leaving the provision of services in their hands.^16,17^

However, conscientious objection is also included both in the law and clinical standards as a provider’s right in line with medical ethics. Except if the absence of care can cause irreversible health damage or threaten the woman's life, healthcare providers shall exercise their professional duties with broad autonomy, not being obliged to provide services that put them in conflict with their cultural, moral, or religious beliefs. In cases where the objection fits, women’s rights to access abortion services shall not be denied, and referral to another health professional in the same or another health facility must be assured.^15,16^

Acknowledging the importance of laws, policies, and supplies as essential conditions for abortion provision, midwives remain critical in ensuring service delivery. Previous studies in countries that underwent legal reform demonstrate persisting challenges: beliefs among midwives, religious factors, sociocultural norms, and health system deficiencies.^18–21^ In particular, the sociocultural, political, and economic context in which reproductive health care is delivered matters, playing a significant role in shaping specific challenges.^22^

Previous studies following the legal reform in Mozambique have largely focused on women’s perspectives and experiences. They have examined how women’s abortion decision-making and access to services are shaped by individual, social, and structural factors, including limited autonomy, poor knowledge of the law, stigma and moral judgements, and gender norms leading to partner and family influence. Additionally, socio-economic determinants such as education, wealth, employment, and marital status, underscoring economic inequality, constrained access to safe services, often resulting in delays or recourse to unsafe abortion pathways.^23–28^

In contrast, provider perspectives remain largely unexplored, particularly regarding how providers’ experiences influence service provision for young women, who are disproportionately affected by abortion-related mortality.

This study explores experiences and responses among midwives concerning the provision of legal abortion services to young women,^15–24^ in northern Mozambique.

## Methods

### Study setting

In the Mozambican population of about 34 million people, 54% are under the age of 18. The median fertility rate is 4.9. This study was conducted in Nampula Province, the most populous among the 11 Mozambican provinces, housing 20% of the national population. About 70% of Nampula inhabitants rely on rudimentary subsistence agriculture. The 52% illiteracy rate among those 15 years old and above perpetuates intergenerational poverty.^29^ Poverty, affecting 70% of its population, is exacerbated by frequent cyclones, disproportionately affecting those reliant on farming for food and income. Traditional practices, such as bride-price, initiation rites, and polygamy, deeply ingrained in societal norms, often reinforce the practice of early and forced marriage as a means of alleviating family poverty. Difficulties in accessing health services (36%), low contraceptive use (13%), and high unmet need for contraception (32.2%), heighten vulnerability to early and unintended pregnancies. High adolescent pregnancy (42%) and motherhood among young women aged 15–19 (61%) in Nampula reflect deep social and structural influences on young women’s power, agency, and autonomy over their sexual and reproductive rights, including access to safe abortion.^30^

### Study design

A qualitative phenomenological study was conducted to understand the lived experience among midwives in providing abortion services to young women.^15–24^ Nampula Province was intentionally chosen as one of the first provinces where the Ministry of Health started implementing abortion services after the legal reform. Three districts, one per region, were randomly selected from the 23 districts in Nampula: Erati (northern), Ribaue (central), and Angoche (southern). Two district health facilities were purposefully included in the study to reflect different levels of service provision: a randomly chosen remote primary health centre and a referral facility (District or Rural Hospital) per district. In total, six primary care health facilities were included.

### Study participants and data collection procedures

Midwives working in gynaecology and maternity wards, with at least six months of experience in the selected facilities, were purposefully invited to share their experiences of abortion service provision. The number of participants was guided by midwife availability, willingness to participate, and data saturation. All available midwives were interviewed in the remote health facilities. On the other hand, only midwives on duty in referral hospitals during fieldwork were interviewed. The interviewer stopped recruiting additional midwives once data saturation was reached, meaning that no new information or insights were emerging. With no refusals, a total of ten midwives from three remote and three referral health facilities were interviewed.

### Data collection procedures

The principal investigator conducted face-to-face in-depth interviews in Portuguese, from June to September 2021, at a time and place chosen by the midwives, generally at the health facility, in a place with audio-visual privacy. A research assistant simultaneously took written notes. A pre-tested in-depth interview guide, starting with an open-ended question on their socio-demographic and professional background, facilitated dialogue engagement. Subsequently, specific questions explored midwives’ experience with abortion service delivery, ultimately focusing on service provision to adolescent girls and young women aged 15–24. Probing questions encouraged deeper reflection and insights into their lived experience. The interviews, lasting 45–60 minutes, were audio-recorded. The authors held discussions after interviews and made key notes on the main findings, but also had meetings during analysis to discuss the interpretation of data.

### Data processing and analysis

Digitally recorded interviews were transcribed verbatim in Portuguese. To ensure accuracy, a team member, not involved in data collection, cross-checked the transcriptions against the original audio recordings. Inspired by Sundler et al, thematic analysis rooted in descriptive phenomenology was applied.^31^ Sundler’s approach articulates Braun and Clarke’s thematic analysis within Russell’s phenomenological study design, allowing a structured yet immersive exploration of narratives. The principal investigator and one co-author not involved in data collection independently coded the data manually. Inductive coding generated categories and themes reflecting the meaning attached to midwives’ experiences with abortion provision. Codes and themes were discussed until consensus was reached. Providing empirical evidence, de-identified quotes were translated into English. The team practised continuous reflexivity and adherence to the revised guidelines: Consolidated Criteria for Reporting Qualitative Research (COREQ).^32^ Grounded in Kimberlé Crenshaw,^33^ the intersectionality approach adopted by Abrams et al^34^ facilitated discussion and interpretation of the findings during analysis.^33,34^ This framework was particularly suited to uncover the nuanced ways in which midwife experience is shaped, and how, in turn, this informed their response to abortion provision, considering the following intersectionality principles: (1) individuals possess multiple intersecting identities; (2) these identities are contextually positioned within systems of power, privilege, or oppression; and (3) sociocultural and structural forces shape their perceptions, experiences, and response to care provision.^34^

### Scientific and ethical considerations

Scientific and ethical approval was obtained from the Institutional Bioethics Committee for Health at the Faculty of Medicine, Maputo Central Hospital (CIBSFM/HCM), reference number CIBSFM&HCM/122/2019, approved on 30 March 2020. Administrative authorisation was secured at the national, provincial, and district levels. No prior relationship was established between the research team and the participants before the commencement of the study. An information sheet detailing the study objectives, data collection procedures, and voluntary participation was made available at health facilities before data collection and further explained at the beginning of each interview. Written consent was obtained from all informants. They were informed of their right to withdraw at any time without consequences. CIBSFM/HCM contact information was provided for any study-related concerns. Given the sensitivity of the topic, the interviewer paid close attention to visual emotional distress. Had it appeared, the interview would have been interrupted. Confidentiality was ensured by storing the consent forms in a secure location, separate from the data. Additionally, interview transcripts were de-identified and assigned a code number, avoiding linkage of data with names.

### Author reflexivity

GGS, the principal investigator, is a female Mozambican researcher with a background in social science and experience in sexual and reproductive health, and a particular interest in adolescent sexual and reproductive health and rights. KM and EM are both Mozambican female researchers in the field of social and medical anthropology, with extensive experience in sexual and reproductive health and qualitative research. BE, PA and JB are female Swedish gynaecologists and obstetricians based in Sweden, with experience in sexual and reproductive health. The research team remained aware that their contextual knowledge, individual and professional backgrounds could potentially influence data collection and analysis. However, having external co-authors who are providers and Portuguese speakers who did not participate in the data collection helped to critically assess the interpretation of data.

## Results

### Description of the study participants

The 10 interviewed midwives were all Christian females, aged 29–50. Most had technical diplomas (mid-level) training in maternal and child health. Two midwives held university-level nursing qualifications. Six midwives worked in hospitals, located in the district headquarters, four in health centres in remote areas. Work experience varied from 2 to 18 years. Seven midwives had over 10 years of experience.

### Themes

Codes were first generated inductively from the data and then grouped into categories and preliminary themes. As themes were refined, we observed that participants’ accounts consistently highlighted tensions and trade-offs, such as between professional obligations and personal beliefs, legal provisions and social norms, and young people’s rights and parental authority. These tensions cut across all categories and themes. To capture this overarching pattern, we developed the theme “navigating dilemmas” representing the central thread connecting participants’ experiences. The overarching theme of navigating dilemmas in providing legal abortion supports two main emerging themes: structural dilemmas and personal dilemmas. Midwives emphasised four main structural dilemmas: (1) a limited number of accredited health facilities, (2) difficulties in referrals, (3) medicines out of stock, and (4) legal requirements for adolescents under 16. At the personal level, they emphasised three dilemmas: (1) how professional duties conflict with moral and religious values, (2) the physical and emotional burdens of providing abortion services, and (3) social norms towards adolescent sexuality.

### Structural dilemmas

#### Limited number of accredited health facilities

Despite abortion being legal, midwives emphasised how structural factors shaped their ability to provide services. Among the health facilities included in this study, only hospitals were accredited by the MoH to provide abortion services. This limitation consistently emerged in their narratives in two main ways: overburdening hospitals with cases that could be handled at health centres, and restricting women’s access to services by preventing service provision at their nearest facility. A midwife shared her point of view, also shared by many of her colleagues:

“… *The rural hospital is the only health facility in the district, out of nine, authorized to offer induced abortion services. Health centres must refer women seeking abortion to us, despite having a nurse and maternity ward … now people know abortion is legal and seek services. Reaching a health facility with obstetric care and not being provided with such service is pointless * … ” (Midwife 7, hospital)

All the midwives expressed support for the expansion of abortion services to remote health facilities as crucial in preventing unsafe abortion and related consequences. Their support was rooted in the belief that abortion, particularly using the combination mifepristone-misoprostol regimen, is a safe and effective life-saving procedure, simple to administer, and suitable for any healthcare facility with a maternity ward and a midwife:

“* … I’ve seen enough women dying from unsafe abortions … Offering at least pharmacological abortion in remote health facilities would prevent unsafe abortions and save women’s lives, especially teenagers … *” (Midwife 6, hospital)

#### Difficulties in referrals

Other structural barriers affected the midwives’ ability to facilitate women’s access to abortion services, in line with MoH guidelines: long distances between remote and referral health facilities, and lack of facility-based transport. A midwife said: *“We don’t have fuel or transport to pick them up, even for obstetric emergencies, it’s been hard  * … *”* (Midwife 9, health centre). The severity of the transportation issue, coupled with patients’ inability to afford private options, significantly hindered referrals, often leading to women resorting to unsafe abortions and returning to health facilities with complications. A midwife from a facility without abortion services elaborated on referral challenges:

“*Since we don’t provide induced abortion here, we must refer to a Rural Hospital, but very few end up going, alleging transportation costs … Many live 50 kilometres away from this health centre, then, another 30 kilometres to reach rural hospital, is hard … They go home and use traditional methods. When facing complications, they come back, while days before they were here seeking safe abortion * … * **We must deal with complications that could have been prevented, if, safe abortion had been provided … so, what are we doing?*” (Midwife 8, health centre)

#### Medicines out of stock

A major barrier to providing abortion services includes *medicines out of stock*. Within accredited health facilities, midwives consistently identified recurrent stock-outs of the combination regimen of *mifepristone* *+* *misoprostol*, introduced after the legal reform, as a major barrier to providing abortion services:

“*For the last 4 months, we have been without mifepristone + misoprostol in the district. Even the province doesn’t have. I believe it is a national stockout. Since then, we have not been providing induced abortion services … *” (Midwife 1, hospital)

Although in the absence of a combination regimen, misoprostol alone can be used, most providers expressed discomfort in doing so. They voiced concerns about the need for multiple administrations, and prolonged monitoring requiring women to remain under supervision until treatment effects were confirmed.

One midwife supported the use of a single regimen, saying: “*I use misoprostol on its own to not let them go home without any service provided, then trying unsafe methods * … *”* (Midwife 3, hospital). However, they generally perceived that inefficiency of misoprostol, compared to the combination regimen, leads to a higher workload. The same midwife explained:

“* … miso + mife is very effective. It doesn’t fail at all, and the patient doesn’t need to remain at the hospital. But when only misoprostol is used, the patient may stay for 24 hours, as often there is induction failure. Sometimes you need to induce it 2–3 times, until the situation is resolved … *” (Midwife 3, hospital)

Consistent with the perception that the combination regimen offers a less burdensome experience for both patients and healthcare providers, midwives at accredited facilities often provide prescriptions to pregnant women to purchase the combination drug at private pharmacies when the combination drug is unavailable. Otherwise, the women seeking an abortion are sent back home without services. In a sad tone, a midwife described how such a situation put them in a difficult position:

“… *as we don’t have mifepristone + misoprostol combined regimen, we prescribe women to buy it at the private pharmacy. Those who cannot afford it just go home … it’s hard for us seeing a woman walking away with no other choice than keeping an unwanted pregnancy or trying other ways by herself. But what can we do?*” (Midwife 2, hospital)

#### Legal requirements for adolescents under 16

Generally, midwives held favourable views towards providing abortion services to adult women (18 years and older). However, their approach shifted noticeably when it came to adolescents under 16. The challenges in serving this group stemmed from the legal requirement mandating the presence of an adult witness. According to the midwives, many adolescents are unwilling to comply with this requirement, and the reluctance of adolescents to present themselves with an adult witness limits the midwives’ ability to provide abortion services to this specific age group:

“* … if that happens, I don’t take risks, I don’t provide services … .*” (Midwife 1, hospital)

“*We are talking about children; we must comply with the law … !*” (Midwife 3, hospital)

Furthermore, since the law requires adult consent, fearing consequences, midwives tended to concur that adults are in a better position to make such decisions.

“* … if the witness does not consent, we do not provide induced abortion to adolescents, as they can make a case complaining about it.*” (Midwife 2, hospital)

While midwives consistently emphasised adult witness consent as a non-negotiable legal requirement for their profession, they nevertheless acknowledged its potential negative consequences, such as adolescents resorting to unsafe abortions. However, they also stressed that in case of an adverse event of any kind (medical, psychological, or social), the adult witness consent serves as a legal protective factor for midwives, as reflected in the following quote:

“* … it’s embarrassing and difficult for them to bring a witness, but they have to … They seek abortion in secret and want to keep it that way, but the law only protects the provider if an adult witness gives consent, we have to comply with it … some come back with someone, others don’t … *” (Midwife 3, hospital)

Some midwives extended the requirement for an adult witness beyond the legal stipulation, applying it to post-abortion care and to adolescents under 18 instead of under 16. Citing concerns about potential professional and legal repercussions, they explained that an adult witness for post-abortion care was necessary to confirm that the termination had been initiated at home. A midwife says:

“* … families can sue the midwife, alleging that she did it without the consent of the relatives, while legally being a child. So, we ask the family to make a written statement, saying it was initiated at home. We have never had such case, but it’s always good to have something for protection. You never know tomorrow.*” (Midwife 5, health centre)

### Personal dilemmas

#### Self-moral and religious dilemmas

Personal dilemmas’ foremost concern is professional duties conflicting with moral and religious values. Midwives cited the religious principle “*no one has the right to terminate their own or another’s life”* (Midwife 7, hospital) as a fundamental reason for their moral distress and unwillingness to provide abortion services. Non-objecting midwives expressed the moral dilemma, feeling complicit, considering what their colleagues perceived as unacceptable and wrongful (e.g. committing murder, ending a potential life, sinful) while fulfilling professional duties. This tension deeply influenced their experience in providing abortion services. A midwife expressed her feelings:

“*While others claim objection, for us already on the path of sin, there is no way to turn back … the professional responsibility has already buried me. There were times, when I would spend days without sleeping, thinking I took a life, why didn’t I refuse? It weighed heavily on my conscience … but … what to do? it’s part of my duties.*” (Midwife 10, hospital)

#### Physical and emotional burdens of providing abortion services

Although religious and moral values were most frequently cited as the primary reason for objections to providing abortion, distressing encounters with severe complications and fatalities deepened the midwives' moral concerns. Deeper reflection on religious values and beliefs surrounding abortion brought attention to how these were often closely linked to past traumatic experiences managing complications from unsafe abortions, including the loss of young lives. A midwife with extensive work experience, who objected to abortion on religious grounds, shared her experience, also shared by another midwife, highlighting negative events related to abortion:

“* … Many women died from abortion. I have seen it. There are cases that I still have in my mind … these things marked my mind negatively. If a woman’s life is in danger, I can provide services, but just because you didn’t know what you were doing, got pregnant, and out of nowhere, you want to terminate it, no. I will refer to someone else … *” (Midwife 7, hospital)

Such objecting views create a physical and emotional burden among the non-objecting midwives. A midwife in her second year of work experience, who did not express objection, described herself feeling overwhelmed by being the only one delivering abortion services at her health facility. She articulated her sense of burden and unfairness in bearing sole responsibility for abortion services, stating:

“* … When a woman comes, they say to me “do it”, I can’t. Even when I am not on duty, they may call me and ask if I am around. I’m already feeling bad and tired, so I end up refusing. Is it just me who must do it? Perhaps it’s worth that I’m not doing it either … I am also giving up.*” (Midwife 4, hospital)

#### Social norms towards adolescent sexuality

Another key factor shaping midwives’ professional stance on abortion provision for adolescents under 16 concerns social norms concerning adolescent sexuality. It was, however, noteworthy that, unlike with adult women, midwives perceived that young girls seeking an abortion should be subjected to scrutiny. This approach appeared to be rooted in societal norms and expectations, but also in the regulation on the age of consent for sex at 16. In other words, by law, sex under the age of 16 is unlawful; thus, socially, you are not expected to be sexually active. A midwife says:

“* … if a mother coming to seek induced abortion service, it’s because she really needs it … for under 16, we investigate why she wants to terminate the pregnancy and we call the family to help us clarifying what happened, why it happened, and what kind of case we are dealing with … *” (Midwife 6, hospital)

#### Views on abortion provision

The midwives all expressed support for the expansion of these services to remote health facilities as crucial in preventing unsafe abortion and related consequences. Their support was rooted in the belief that abortion, particularly using the combination regimen, is a safe and effective life-saving procedure, simple to administer, and suitable for any healthcare facility with a maternity ward and a midwife:

“* … I’ve seen enough women dying from unsafe abortions … Offering at least pharmacological abortion in remote health facilities would prevent unsafe abortions and save women’s lives, especially teenagers … *” (Midwife 6, hospital)

## Discussion

This study explored the lived experience among midwives in providing legal abortion services to young women in Nampula province, northern Mozambique. Our findings align with previous studies, indicating that midwives worldwide face multiple challenges when providing legal abortion care.^35^ Previous research often emphasises conflicts between professional duties and moral or religious beliefs as key intersecting factors shaping the unwillingness and objection to providing abortion services among midwives.^36,37^ While we also found this to be a prominent barrier, our findings suggest that distressing professional experience dealing with and coping with severe complications from unsafe abortion plays a significant role. Exposure to severe patient complications and consequent disengagement in providing care is widely documented in chronic disease care.^38^ However, the role of such encounters as primarily shaping unwillingness to take part in abortion provision remains underexplored. Some previous studies emphasised other factors, including histories of abortion, miscarriage, or parenting in challenging circumstances, playing a role in midwives’ motivation for abortion service provision.^39^

Objection towards service provision following exposure to severe cases of complications was predominant among senior midwives working in hospitals, often holding leading positions. However, one senior midwife working in a hospital expressed support for providing abortion – even when only misoprostol was available. This suggests that associating severe abortion-related complications with attempted unsafe abortions due to limited access to services can also lead to motivation to provide the service, in order to prevent pregnant women from seeking unsafe alternatives. A related finding emphasises the experience and response among younger midwives. While working in hospitals, they did not refer to traumatic experiences with abortion-related complications. Nevertheless, they reported occupational distress from an increasing workload, caused by the objecting senior colleagues, leading to reluctance to continue providing services.

Divergent views and practices among midwives regarding abortion service delivery have been observed in other settings.^40,41^ Our study suggests that there is no fixed line between being willing and objecting to providing services. Midwives who have been providing services may become objectors. Factors driving the shifting arguments include encounters with severe complications, a lack of support with coping mechanisms, pressure, and workload. This also underscores a crucial point: while exposure to severe complications may influence lived and perceived experience, different intersecting factors may play a role in determining midwives’ stance and response towards abortion provision. The findings in this study support this idea. For example, while being older, more experienced, and in a leadership role seemed to place midwives in a privileged position to enforce their right to conscientious objection, being younger and less experienced places others in an oppressive position, carrying the responsibility of offering the services alone.

The intersectional framework highlights how sociocultural and structural forces shape perceptions, experiences, and responses to care provision, in line with our findings. The current legal framework strongly shapes the responses to abortion provision among midwives. While acknowledging that the challenges under-16 adolescents face in accessing abortion services are different from those facing adult women, and that service denial could drive them towards unsafe abortions, how midwives strictly adhered to the legal age-requirements is concerning. Following the law provides legal legitimacy to midwives’ actions, granting adult witnesses the decision-making power and authority over abortion choices among adolescents. However, this may reveal a misinterpretation of the legal framework, which grants decision-making rights solely to the pregnant individual, regardless of age. The interpretation and indiscriminate use of age-related legal requirements among midwives – not only for those below 16 but also below 18 – seemed to be intersecting with prevailing social norms towards adolescent sexuality, which is often viewed as inappropriate. These perspectives further illustrate how social norms drive negative responses to abortion provision and reinforce power dynamics that can inadvertently restrict adolescent autonomy.

Conflict between adolescent sexual and reproductive rights and societal norms has been reported in previous studies conducted elsewhere,^35,42^ including the legal requirements for adult witnesses preventing timely care-seeking and access to abortion services.^43^ Recently conducted systematic reviews on the impact of parental involvement laws on minors seeking abortion services, globally^43^ and on abortion among adolescents in Africa,^44^ emphasised how adult involvement laws can potentially decrease abortion rates, as they may also lead to “forced” continuation of unintended pregnancies, and increased teenage birth rates as well as delayed or unsafe procedures.^44^ Such consequences may ultimately constitute violations of reproductive rights.

Our findings corroborate that there is a complex interplay between legal frameworks and social norms and illustrate how this may potentially override young girls’ autonomy over their sexual and reproductive rights. This interplay has been overlooked. Mozambique’s penal code establishes 16 as the age of consent for sexual relations. This may possibly be the source of heightened scrutiny of abortion care seekers below this age. However, the high rates of unintended pregnancy among adolescents, early motherhood, and child unions in Mozambique^45^ highlight the need to reassess whether the national abortion services and related laws and policies adequately address adolescent needs and support midwives with their duties of ensuring women’s rights to abortion services. In this regard, Ferguson and colleagues emphasise that some countries are unprepared for addressing adolescent reproductive needs, due to ageist and protectionist social norms, resulting in laws and policies that often fail adequately to address such needs.^46,47^ Restricting access to safe abortion for those under 16 contradicts evidence on adolescent sexual and reproductive health in the country^48^ and may potentially undermine the legal reform’s intended impact.

Finally, while stock-outs of abortion medication emerged as a major factor preventing midwives from providing services, as reported in a scoping review,^49^ midwives never referred to the use of surgical abortion, despite this being a legal option. Rather, they advised women to purchase combination drugs privately. While this may reflect an effort to mitigate stock-outs and the midwives’ commitment to provide services, it simultaneously suggests midwives applying preferences for pharmacological over surgical methods in managing abortion, while medicines remain unavailable. Prioritising pharmacological methods has been reported in Europe, Southeast Asia, and Sub-Saharan Africa.^21,50^ Nevertheless, our findings suggest that while midwives resort to the private sector as a strategy for granting access to and provision of abortion care, this solution creates a significant dilemma. It becomes an option only for those who can afford it, leaving those who cannot without a service that is legally free of charge. Nevertheless, being unable to provide care to those who cannot afford it leads to professional distress among midwives, forcing them to navigate complex moral and ethical dilemmas shaped by structural and systemic limitations beyond their control.

Policy efforts should address these barriers. Expanding accreditation to all primary healthcare centres with a maternity ward is crucial to guaranteeing safe, effective, and less burdensome care for both patients and providers. Primary healthcare centres, particularly in remote areas, should be able to at least offer combination regimen therapy. Efforts should prioritise ensuring consistent availability of medical abortion medications and supporting their safe, client-centred use. Strengthening supply chains to prevent stock-outs and maintaining provider readiness are essential to meet women’s preferences and reduce reliance on unsafe alternatives. Policies regarding adolescent access to abortion should be aligned with human rights standards, ensuring that the autonomy of pregnant adolescents is respected, while safeguarding providers from legal repercussions. Finally, training and continuous support for midwives, including addressing traumatic experiences, should be prioritised to strengthen resilience and service delivery.

Overall, revisiting the legal, health system, and professional support structures is essential towards realising the full benefits of the 2014 abortion law in ensuring providers/midwives’ response towards safe abortion provision, reducing preventable maternal deaths among young women in Mozambique.

### Strengths and limitations of the study

To the authors’ knowledge, this is the first published phenomenological study examining midwives’ experiences with abortion provision specifically for young women, following the legal reform in Mozambique. The findings offer contextual insights on how provider experience, coupled with the system’s capacity and legal requirements, shapes their attitudes and responses towards service provision for young women, with a particular impact on adolescents. While findings may inform the legal framework in Mozambique and beyond, the transferability and confirmability to other Mozambique provinces and beyond may be limited. Mozambique is a culturally diverse country, and Nampula province has unique demographic characteristics. Being the most populated province, with limited resources compared to other provinces, may interfere with the capacity for service provision. Therefore, the results may not represent the national context and should be interpreted specifically within the provincial context.

## Conclusion

Regardless of personal or moral stances, midwives in northern Mozambique largely support the principle of providing abortion services. However, their ability and willingness to deliver these services are significantly shaped by structural and personal dilemmas and professional distress. Midwives are particularly constrained by a limited number of accredited facilities, frequent stock-outs of essential medications, burdensome legal requirements for adolescents under 16, and the emotional impact of past traumatic experiences. These challenges not only affect their work satisfaction and mental health, but moreover jeopardise the quality and accessibility of abortion services, especially for adolescents, a group already at high risk of unsafe abortion and maternal mortality. As such, policy efforts should focus on reducing barriers to safe abortion care, expanding accreditation to all primary healthcare centres with maternity wards, especially in remote areas, to provide at least a combination regimen therapy that can ensure safer, more accessible services. Adolescent access policies should be aligned with human rights, respecting their autonomy while protecting providers from legal risks. Strengthening midwives’ training and support, including addressing traumatic experiences, is also essential.

## Supplementary Material

Supplemental Material. VTP provision by level of care and healthcare cadres (15,16).

## Footnotes

* *Interrupção voluntária da gravidez* (IVG) is used in Mozambique to refer to an induced abortion.

## Provenance statement

This article was not commissioned and went through external peer review.
