# Supplementary material for: Navigating structural and personal dilemmas: a phenomenological study of midwives providing legal abortion care in Nampula, Mozambique
Source: Sex Reprod Health Matters. 2026 Mar 4;33(1):2637328. doi: 10.1080/26410397.2026.2637328 (PMC13072679; doi:10.1080/26410397.2026.2637328)
Supplement: Supplemental Material. VTP provision by level of care and healthcare cadres (15,16). [file ZRHM_A_2637328_SM1096.docx]

**Supplement**

VTP provision by level of care and healthcare cadres (15,16)

| LEVEL OF CARE | TYPE OF HEALTH FACILITY | PROVIDED VTP SERVICE | CADRES PROVIDING VTP SERVICES |
| --- | --- | --- | --- |
|  |  |  |  |
| Primary health care | Health centres type II | Pharmacological VTP up to 12 weeks’ gestational age;  Complications shall be referred to the following level of care | Midwives/maternal and child health nurses |
|  | Health centres type I | Pharmacological + surgical VTP up to 12 weeks’ gestational age;  Complications shall be referred to the following level of care | Midwifes/maternal and child health nurses, mid-level general practitioners (technicians), physicians (general practitioners) |
| Secondary level of care | District and Rural Hospitals | Pharmacological and surgical methods can be provided and abortion-related complications can be treated | The cadres above + **surgical technicians (**a distinct mid-level cadre trained to perform surgeries), graduate nurses in maternal and child health care |
| Tertiary level of care | General and provincial hospitals | Pharmacological and surgical VTP up to 12 gestational age, cases falling up to 16 and 24 weeks and treatment of severe complications. | Cadres above+  Obstetrician-Gynaecologists |
| Quaternary level of care | Central hospitals |  |  |
